# Continuous and batch cultures of *Escherichia coli* KJ134 for succinic acid fermentation: metabolic flux distributions and production characteristics

**DOI:** 10.1186/1475-2859-12-80

**Published:** 2013-09-17

**Authors:** Carel D van Heerden, Willie Nicol

**Affiliations:** 1 Department of Chemical Engineering, University of Pretoria, Hatfield, Pretoria 0002, South Africa

**Keywords:** *Escherichia coli* KJ134, Succinic acid, Metabolic flux distribution, Chemostat, Growth inhibition, Non-growth production, D-glucose, Mineral salt medium

## Abstract

**Background:**

Succinic acid (SA) has become a prominent biobased platform chemical with global production quantities increasing annually. Numerous genetically modified *E. coli* strains have been developed with the main aim of increasing the SA yield of the organic carbon source. In this study, a promising SA-producing strain, *E. coli* KJ134 [Biotechnol. Bioeng. 101:881–893, 2008], from the Department of Microbiology and Cell Science of the University of Florida was evaluated under continuous and batch conditions using D-glucose and CO_2_ in a mineral salt medium. Production characteristics entailing growth and maintenance rates, growth termination points and metabolic flux distributions under growth and non-growth conditions were determined.

**Results:**

The culture remained stable for weeks under continuous conditions. Under growth conditions the redox requirements of the reductive tricarboxylic acid (TCA) cycle was solely balanced by acetic acid (AcA) production via the pyruvate dehydrogenase route resulting in a molar ratio of SA:AcA of two. A maximum growth rate of 0.22 h^-1^ was obtained, while complete growth inhibition occurred at a SA concentration of 18 g L^-1^. Batch culture revealed that high-yield succinate production (via oxidative TCA or glyoxylate redox balancing) occurred under non-growth conditions where a SA:AcA molar ratio of up to five was attained, with a final SA yield of 0.94 g g^-1^. Growth termination of the batch culture was in agreement with that of the continuous culture. The maximum maintenance production rate of SA under batch conditions was found to be 0.6 g g^-1^ h^-1^. This is twice the maintenance rate observed in the continuous runs.

**Conclusions:**

The study revealed that the metabolic flux of *E. coli* KJ134 differs significantly for growth and non-growth conditions, with non-growth conditions resulting in higher SA:AcA ratios and SA yields. Bioreaction characteristics entailing growth and maintenance rates, as well as growth termination markers will guide future fermentor designs and improvements.

## Background

Succinic acid (SA) is poised to become a major bulk-scale biobased chemical. The projected annual commercial production of ‘biosuccinic acid’ at the end of 2015 is estimated to be in excess of 150 ktons per annum, with five separate companies (BASF-Purac, Bioamber, Reverdia (DSM-Roquette), Mitshubishi-PPT and Myriant) currently developing and implementing pilot- and industrial-scale plants [1,2]. Although traditionally seen as a speciality chemical, new applications like the production of polybutylene succinate to manufacture biodegradable plastic have stimulated research on, and the development of, fermentative SA production. SA can also replace petrochemical building blocks to produce intermediate chemicals like tetrahydrofuran, γ-butyrolactone, 1,4-butanediol and maleic anhydride. Various reviews are available on the general topic of producing SA with biocatalysts [3-9]. Additionally, reviews by Beauprez et al. [10] and Cheng et al. [11] focus specifically on genetically modified microorganisms. Cukalovic et al. [7] focus on the viability of industrial biochemical SA production, while providing an overview of the current petroleum process. The most reported SA-producing species include *Actinobacillus succinogenes*, *Mannheimia succiniciproducens, Anaerobiospirillum succiniciproducens* and various strains of modified *E. coli*[4,10]. These bacteria, in addition to recombinant *Corynebacterium glutamicum*, have been identified by McKinlay et al. [5] as the most promising SA producers. Limited development on yeast strains has also been reported, with low pH fermentation the main objective [2,12,13].

The open literature reports that the highest yields are achieved by modified *E. coli*[14-18]. These yields are in excess of 1 g SA per g glucose consumed (the theoretical maximum is 1.12 g g^-1^). All studies employed batch or fed-batch fermentation, with some utilising initial aerobic growth of cells [10,18]. From a bioreaction engineering perspective, these promising strains require further exploration. This will allow the development of novel fermentor designs to optimise productivity and product titre. Given the projected scale of SA production, these will be key factors in the economic viability of the biobased process. In this regard the kinetic behaviour of the cells is important, and growth and maintenance rates, as well as inhibition characteristics, need to be quantified. It is also important to quantify the distribution of metabolic products as a function of the state of the cell (growing or non-growing) as this will affect the overall yield [19-21].

In the laboratory, batch or fed-batch fermentors are preferred over continuous fermentors. The reason for this is mostly a practical one, as batch or fed-batch systems are not subject to the experimental challenges of continuous operation.. Successful batch/fed-batch-based processes from the laboratory are often directly scaled up to commercial units in order to obtain the desired product. Up-scaling entails numerous engineering challenges, and optimality cannot be guaranteed since the process is essentially a larger copy of the laboratory unit. For small-scale fermentors (producing high-value products) process optimality is typically not the main economic driver. However, large-scale fermentation (for bulk chemical production such as SA) is different, as the efficiency of production is crucial to the economic success of the process. Continuous production has numerous advantages over conventional batch/fed-batch production, where higher productivities reduce capital expenditure and prolonged operation reduces operating costs. Major disadvantages include possible strain mutation over time and inflexible operating conditions. Also, infections can cause upsets in the operation of the whole plant. Continuous conditions are superior for studying metabolic flux distribution, where steady state allows proper mass balances to quantify fluxes accurately [22,23]. Accordingly, continuous conditions are ideally suited for studying the performance characteristics of the microorganism.

In this study one of the promising high-yield *E. coli* strains, KJ134, from the group of Prof. L. Ingram from the University of Florida was investigated. A yield of 1 g g^-1^, with a final SA titre of 71 g L^-1^ using a minimal medium in a batch fermentor has been reported. All genetic modifications of the bacterium are given in Table 1[14,24]. *E. coli* KJ134 is evaluated under chemostat and batch conditions while continuous CO_2_ (g) sparging was used for supplying inorganic carbon. Growth and maintenance characteristics are quantified in terms of rate, inhibition and product distribution in order to provide a basis for optimal fermentor design.

**Table 1:** **Gene modifications to *E. coli *C to obtain the succinate-producing *E. coli *KJ134**[14,24]

**Enzyme**	**Modification**	**Abbreviation**
2-ketobutyrate formate lyase	Inactivation	∆* tdcE*
Acetate kinase	Inactivation	∆* ackA*
Alcohol dehydrogenase	Inactivation	∆* adhE*
Aspartate aminotransferase	Inactivation	∆* aspC*
Citrate lyase	Inactivation	∆* citF*
Formate transporter	Inactivation	∆* focA*
Lactate dehydrogenase	Inactivation	∆* ldhA*
Methylglyoxal synthase	Inactivation	∆* mgsA*
NAD^+^-linked malic enzyme	Inactivation	∆* sfcA*
PEP carboxikinase	Overexpression	∆* pck*
Phosphotransacetylase	Inactivation	∆* pta-ackA*
Pyruvate formate lyase	Inactivation	∆* pflB*
Pyruvate oxidase	Inactivation	∆* poxB*
Threonine decarboxylase	Inactivation	∆* tdcD*

## Results

### Continuous fermentations

**Figure 1:**
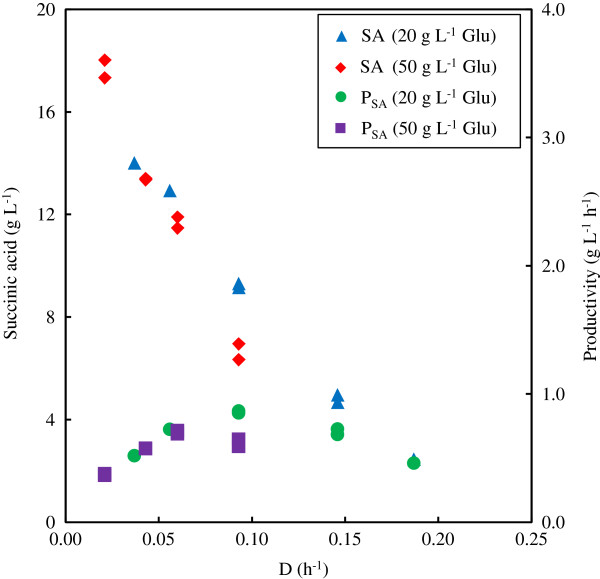
**Steady state results from chemostat fermentations with glucose feed concentrations of 20 and 50 g L**^**-1**^**.** SA effluent concentration and SA productivity given as a function of dilution rate. Glu – glucose; SA – succinic acid.

All steady state results are reported in Table 2. Genetic stability was confirmed by repeating dilution rates at different times during the fermentation (the longest fermentation lasted 29 days). Steady state was confirmed by ensuring a steady dosing rate of potassium hydroxide (KOH) in excess of 10 hours. Figure 1 presents the SA concentration and productivity for all steady states as a function of the dilution rate employed. A similarity between the 20 and 50 g L^-1^ glucose feed fermentations is observed, although the 50 g L^-1^ fermentations appear to be inhibited by the high substrate concentration at a dilution rate of 0.09 h^-1^. This is evident from the start of a decline in productivity between dilution rates of 0.07 and 0.09 h^-1^. The maximum growth rate for the 20 g L^-1^ glucose fermentation occurred at approximately 0.225 h^-1^ (extrapolation to SA concentration of 0 g L^-1^ at washout). The extrapolated value for the 50 g L^-1^ glucose fermentations appears to be slightly less.

**Table 2:** Steady state results from continuous fermentations

**Dilution rate**	**Effective glucose in feed**^**a**^	**Glucose in effluent**	**SA in effluent**	**AcA in effluent**	**Form A in effluent**	**Malic acid in effluent**	**DCW **	**Mass Balance**^**b**^	**SA/AcA **	**Y**_**SP**_^**c**^	**Y**_**SX**_	**P**
**(h**^**-1**^**)**	**(g L**^**-1**^**)**	**(g L**^**-1**^**)**	**(g L**^**-1**^**)**	**(g L**^**-1**^**)**	**(g L**^**-1**^**)**	**(g L**^**-1**^**)**	**(g L**^**-1**^**)**		**(mol mol**^**-1**^**)**	**(g g**^**-1**^**)**	**(g g**^**-1**^**)**	**(g L**^**-1 **^**h**^**-1**^**)**
0.056	19.0	2.2	12.9	2.9	0.3	0	ND^d^	ND^d^	2.3	0.77	ND	0.72
0.146	19.4	12.4	5.0	1.4	0.6	0.38	0.88	1.00	1.9	0.71	0.125	0.73
0.146	19.4	12.7	4.7	1.4	0.5	0.00	0.87	1.00	1.7	0.70	0.130	0.68
0.093	19.0	6.6	9.2	2.5	0.6	0.34	1.97	1.05	1.9	0.74	0.159	0.85
0.093	19.0	6.4	9.3	2.5	0.6	0.00	1.62	1.02	1.9	0.74	0.128	0.87
0.187	19.8	15.8	2.5	0.8	0.3	0.51	0.68	1.00	1.5	0.62	0.171	0.46
0.037	19.0	0.0	14.0	3.9	0.5	0	ND^d^	ND^d^	1.8	0.74	ND^d^	0.52
0.060	49.4	33.9	11.9	3.0	0.5	0.44	1.21	0.99	2.0	0.77	0.078	0.71
0.060	49.2	33.9	11.5	3.0	0.3	0.00	1.21	1.00	2.0	0.75	0.079	0.69
0.093	48.9	39.0	7.0	1.8	0.3	0.37	0.76	0.98	2.0	0.70	0.077	0.65
0.093	49.0	39.6	6.3	1.7	0.4	0.37	1.01	0.98	1.9	0.67	0.107	0.59
0.043	47.9	30.3	13.4	3.5	0.3	0.00	1.41	0.99	2.0	0.76	0.080	0.58
0.043	48.3	30.7	13.3	3.6	0.3	0.54	1.41	0.99	1.9	0.76	0.081	0.57
0.021	47.3	23.5	18.0	4.7	0.8	0.49	1.45	0.98	2.0	0.76	0.061	0.38
0.021	47.3	22.8	17.3	4.4	0.3	0.60	1.53	0.94	2.0	0.71	0.063	0.36

^a^ The dilution effect of KOH dosing incorporated into an effective feed concentration.

^b^ Fraction of accounted mass in effluent (malic acid not used in calculation).

^c^ SA yield based on glucose consumed.

^d^ Not determined.

The SA concentration trend in Figure 1 suggests severe product inhibition. At the lowest practically possible dilution rate of 0.02 h^-1^, the highest SA concentration obtained was 18 g L^-1^ for the 50 g L^-1^ glucose (feed) concentration. Glucose limitations prevented SA concentrations higher than 14 g L^-1^ for the 20 g L^-1^ glucose feed fermentation. The glucose residual at the extreme low dilution of 0.02 h^-1^ suggest that growth terminates around the observed steady state value of 18 g L^-1^ SA. Maintenance production by itself is not possible in a chemostat since outlet biomass cannot be replaced by growth. Growth inhibition is likely caused by all the organic acids, but the available data do not allow distinction between the separate effects of the different acids.

**Figure 2:**
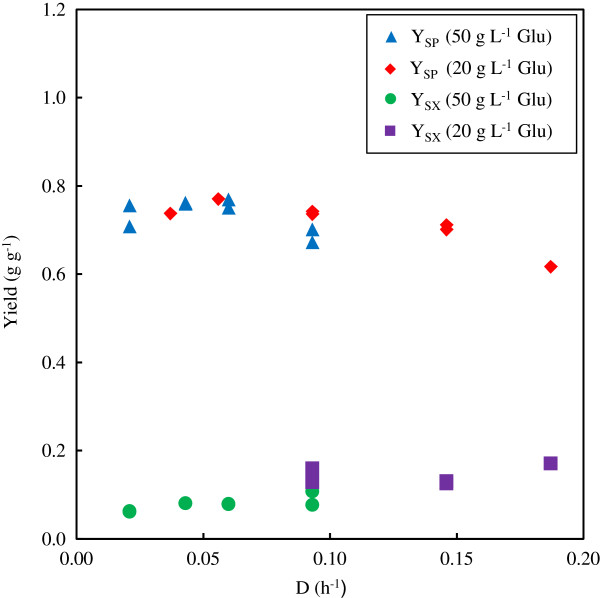
**Yield coefficients (Y**_**SP **_**and Y**_**SX**_**) from chemostat fermentations with glucose feed concentrations of 20 and 50 g L**^**-1**^**.** Glu – glucose.

Figure 2 gives the SA and biomass yields of the continuous fermentations. The SA yields are all between 0.7 and 0.8 g g^-1^, except at the highest dilution rate where low glucose conversions complicate accurate yield determination. Note from Table 2 that the SA to AcA ratio is close to the value of two and that the formic acid amounts are small and considered negligible. The mass balances reported in Table 2 indicate that the measured outlet concentrations account reasonably well for the inlet mass flux, implying accuracy of the reported feed and product measurements.

### Batch fermentations

**Figure 3:**
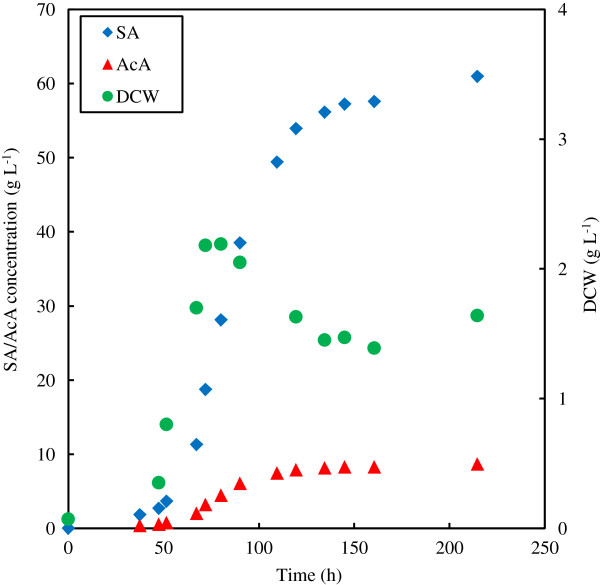
**Concentration profiles of the batch fermentation with an initial glucose concentration of 90 g L**^**-1**^**.** SA – succinic acid; AcA – acetic acid; DCW – dry cell weight.

The results from the batch fermentation employing an initial glucose concentration of 90 g L^-1^ are given in Table 3, with the SA, AcA and dry cell weight (DCW) plotted in Figure 3. An additional batch fermentation was performed with a starting glucose concentration of 50 g L^-1^ (not reported). Very similar initial concentration profiles as that obtained with the 90 g L^-1^ initial glucose fermentation were observed. From Figure 3 the termination of cell growth is observed at an SA concentration of approximately 18 g L^-1^ and an AcA concentration of 3.5 g L^-1^. This is similar to the continuous culture where growth terminated around 18 g L^-1^ SA and 4.5 g L^-1^ AcA.

**Table 3:** **Results of batch fermentation with an initial glucose concentration of 90 g L**^**-1**^

**Time**	**Glucose**	**SA**	**AcA**	**Form A**	**DCW**	**Glucose available**^**a**^	**Y**_**SP**_^**b**^	**SA/AcA**	**∆SA/∆AcA**
**(h)**	**(g L**^**-1**^**)**	**(g L**^**-1**^**)**	**(g L**^**-1**^**)**	**(g L**^**-1**^**)**	**(g L**^**-1**^**)**	**(g L**^**-1**^**)**	**(g g**^**-1**^**)**	**(mol mol**^**-1**^**)**	**(g g**^**-1**^**)**
0.0	90.0	0.0	0.0	0.0	0.07	90	-	-	-
37.5	88.0	1.9	0.4	0.1	ND^c^	90	0.89	2.41	2.4
47.5	87.7	2.7	0.6	0.0	0.35	91	0.79	2.29	2.0
51.5	86.2	3.7	0.8	0.0	0.80	91	0.73	2.32	2.4
67.3	75.0	11.3	2.1	0.2	1.70	89	0.79	2.77	3.0
72.0	64.0	18.7	3.2	0.2	2.18	87	0.83	2.96	3.3
80.0	49.8	28.1	4.5	0.3	2.19	82	0.87	3.18	3.7
90.0	37.3	38.5	6.1	0.5	2.05	81	0.88	3.22	3.3
109.5	20.8	49.4	7.5	0.3	ND^c^	ND^c^	ND^c^	3.35	3.9
119.5	16.7	53.9	7.9	0.2	1.63	75	0.93	3.45	5.1
134.5	13.9	56.2	8.2	0.2	1.45	74	0.94	3.48	4.5
145.0	12.9	57.3	8.3	0.2	1.47	74	0.94	3.50	4.5
160.5	11.8	57.6	8.3	0.2	1.39	73	0.94	3.52	
214.5	9.8	61.0	8.7	0.2	1.64	75	0.94	3.55	4.6

^a^ Glucose present and glucose converted to product (from mass balance).

^b^ Based on calculated glucose available.

^c^ Not determined.

Quantification of the SA yield on glucose is complicated by the dilution effect of NaOH dosing and the continuous removal of samples from the system. A total of 210 mL of 10 M KOH was dosed during the fermentation, and a total of 200 mL of broth was removed as separate samples. The effective amount of glucose available for the fermentation was estimated by performing a theoretical mass balance on each sample (see Table 3). Malic acid was emitted from the mass balance, due to the difficulty of separating the malic acid peak during the HPLC (high-performance liquid chromatography) analysis from the glucose peak when the glucose concentration was high. It is evident that the SA yield starts at a value similar to that of the continuous fermentations (below 0.8 g g^-1^) and thereafter increases to higher yield values (up to 0.94 g g^-1^).

The yield increase is also evident in the SA to AcA ratio. Initially the ratio is similar to that of the continuous fermentations, but it already exceeds two while cell growth is still occurring. In order to analyse the instantaneous production characteristics of the cells, the incremental production ratio of SA to AcA should be considered (see Table 3). At the point of growth termination the incremental ratio is three, while this value increases to as high as five during the maintenance production phase.

The reason for the decrease in DCW in the maintenance production phase is unclear, but was also observed in the (initial) 50 g L^-1^ glucose batch fermentation. The dilution effect due to sample removal and NaOH addition cannot account for the drastic decrease in the biomass content. It is also evident that the maintenance production towards the end of the fermentation is extremely slow, with significant amounts of residual glucose present (10 g L^-1^).

## Discussion

### Succinic acid yield considerations

In order to interpret the measured product distributions and yields, proper analysis of the possible metabolic pathways is required. From an overall perspective the optimum stoichiometry is given by the following balanced reaction where glucose (Glu) reacts with carbon dioxide to form succinic acid (SA):

(1) $$\mathrm{Glu}+\frac{6}{7}CO_{2}\to \frac{12}{7}\mathrm{SA}+\frac{6}{7}H_{2}O$$

The above equation ignores the formation of biomass and gives the maximum possible yield of SA based on one mol of glucose. When expressed in mass units the above equation results in a SA yield of 1.12 g g^-1^.

**Figure 4:**
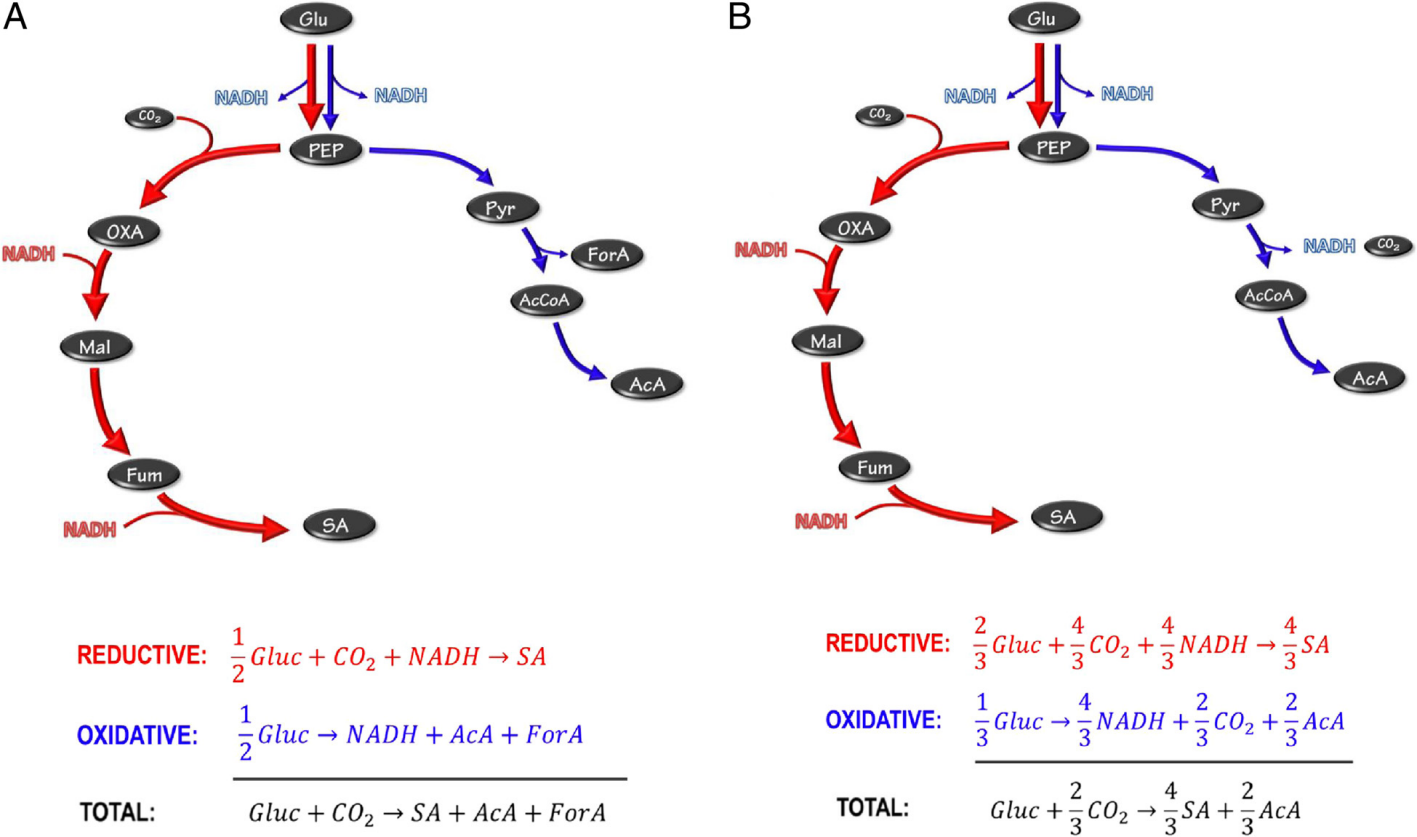
**Metabolic pathways of native SA producers. A)** Pyruvate oxidation via pyruvate formate lyase, **B)** Pyruvate oxidation via pyruvate dehydrogenase. Glu – glucose; PEP – phosphoenolpyruvate; OXA – oxaloacetate; Mal – malate; Fum – fumarate; SA – succinate; Pyr – pyruvate; AcCoA – acetyl coenzyme A; AcA – acetate.

In order to understand metabolic pathways for high-yield SA production, it is useful to consider the pathways of the naturally producing organisms such as *Actinobacillus succinogenes*, *Mannheimia succiniciproducens* and *Anaerobiospirillum succiniciproducens*. All of these organisms produce SA anaerobically via the reverse TCA (tricarboxylic acid) cycle starting with a PEP (phosphoenolpyruvate) carboxylation step. The net stoichiometry from glucose to SA requires NADH (see red arrows in Figure 4) and the pathway is therefore referred to as the reductive pathway. In order to balance the redox an oxidative pathway is required (also starting at glucose as indicated by the blue arrows in Figure 4). All the wild SA producers use AcA as a product to generate the required NADH. Pyruvate can be oxidised via the pyruvate dehydrogenase (PDH) pathway or the formate lyase pathway (FL). In the PDH pathway NADH is released and accordingly the oxidative pathway in Figure 4(B) supplies more reduction power. When balancing the NADH of the two pathways the net oxidative and reductive flux can be determined. The combined overall pathway can then be used to determine the maximum possible yield. For the FL route in Figure 4(A) an equal amount of oxidative and reductive flux occur, implying a 1:1 split of carbon at the PEP node. The maximum mass-based yield for this scenario is 0.66 g g^-1^. For the PFL pathway (Figure 4(B)) the additional NADH formed causes a ratio of 2:1 between the reductive and oxidative paths, thus allowing for more flux to SA where the maximum mass-based yield increases to 0.87 g g^-1^. For this scenario each mol of AcA formed will result in 2 mol of SA formed.

**Figure 5:**
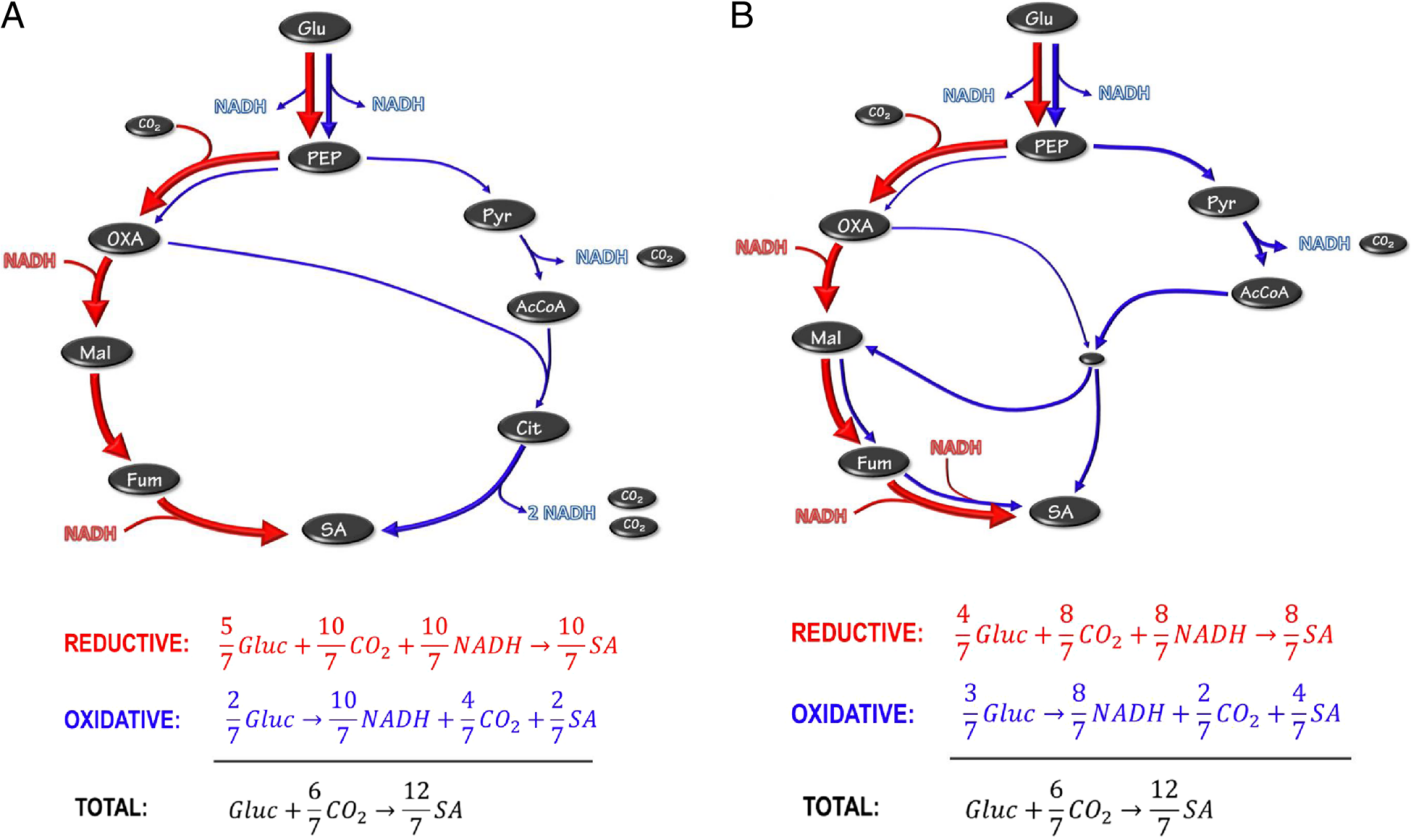
**Metabolic pathway with SA as the only excretion product. A)** Reductive and oxidative TCA sections, **B)** Glyoxylate shunt. Glu – glucose; PEP – phosphoenolpyruvate; OXA – oxaloacetate; Mal – malate; Fum – fumarate; SA – succinate; Pyr – pyruvate; AcCoA – acetyl coenzyme A; Cit – citric acid; AcA – acetate.

It is possible to improve the metabolic pathways of the natural organisms to further enhance SA yields by using an oxidative pathway with SA as the final product. The simplest of these is the oxidative branch of the TCA cycle where reduction power or NADH is generated en route to SA (see Figure 5(A)). The TCA cycle can function in the absence of oxygen, although no NADH can be converted to ATP via oxidative phosphorylation. The major generation of NADH in the oxidative side of the TCA cycle (see Figure 5(A)) causes most of the flux to flow through the reductive part of the cycle (fraction of 5/7 of total flux). It is important to note that the combined stoichiometry is exactly the same as Equation 1. The representation in Figure 5(A) does not dictate termination of the oxidative TCA section at SA. It is also possible to operate the full TCA cycle as the oxidative pathway. In this case the reductive pathway flux will increase while the overall stoichiometry will remain the same as that of Equation 1. The glyoxylate shunt can be employed in a similar manner to achieve the exact same result. The overall oxidative route will share sections of the reductive route. The smaller amount of NADH generated in the oxidative glyoxylate path causes more oxidative flux (fraction of 3/7 of total flux), as indicated in Figure 5(B).

### Analysis of production capabilities and limitations

It is evident that high-yield production of SA by *E. coli* KJ134 occurs under non-growth conditions where AcA production is small. The amount of AcA produced under growth conditions, especially for continuous cultures, resembles that of a native SA producer employing pyruvate dehydrogenase for the oxidation of pyruvate. This is clear for the SA:AcA ratios reported in Table 2. All values are close to the theoretical value of two (see Figure 4(B)) while formic acid production is limited. The chemostat yield values all lie below the theoretical maximum yield of 0.87 g g^-1^ due to biomass formation. The mechanism by which the AcA is produced is unknown, but it is unlikely that it is due to genetic regression. This is because the chemostat fermentations exhibited good repeatability between initial and final steady states after extended operation. For the development of *E. coli* KJ134, numerous minor acetate-forming pathways were deleted [14], with minimal AcA formation reported in the test fermentations.

Product distributions under non-growth or maintenance conditions differ substantially from growth conditions as observed in the batch results. It appears that the metabolic modifications only function after cell growth has terminated, although SA:AcA ratios slightly higher than two were obtained in the growth phase of the batch reactor. The highest SA:AcA ratio of five was obtained in the maintenance phase of the batch reactor, as seen from the instantaneous production ratios in Table 3. It is therefore evident that the ‘ideal’ metabolic pathway represented in Figure 5 is partially utilised under maintenance conditions, where reduction power is generated via the oxidative TCA (or glyoxylate shunt) pathway.

When comparing the batch runs of this study to that of the original [1], the major difference lies in the inorganic carbon source. Jantama et al. [14] employed a mixture of K_2_CO_3_ and KOH for neutralisation and carbonate supply, while continuous CO_2_ (g) sparging was used in this study. Despite a major difference in the initial DCW (0.07 g L^-1^ for this study compared to 0.003 g L^-1^ previously reported), the SA concentration after 96 hours was much higher (71.5 g L^-1^) than in this work (approximately 43 g L^-1^). The SA to AcA ratio was also significantly higher (16) than in this study (4).

The growth inhibition characteristics of the chemostat and batch fermentations agree, where a SA concentration of 18 g L^-1^ (with the associated AcA) can be used as a marker. High-titre production of SA will accordingly only be possible with non-growing cells. The classical fed-batch system is therefore suited for the process. High-titre continuous production will only be viable with a cell recycle system where a separate growth fermentor supplies cells to a cell recycle fermentor. For the proposed scheme AcA production under growth conditions can be bypassed by operating the growth fermentor aerobically. The high SA titre from the non-growing cells will be associated with high SA yields.

**Figure 6:**
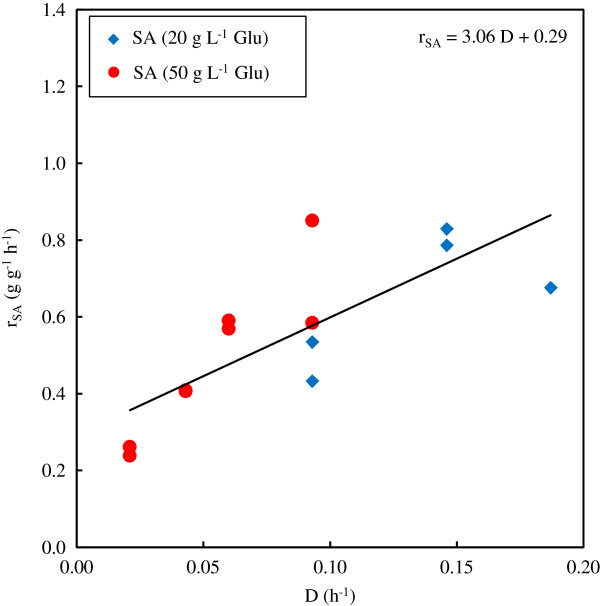
**r**_**SA **_**vs. D for the determination of the maintenance production rate of SA.** Glucose feed concentrations of 20 and 50 g L^-1^ are indicated. Glu – glucose; SA – succinic acid.

Maintenance production rates were quantified for both the chemostat and batch fermentations. The analysis of the continuous fermentations is given in Figure 6 where the cell-based production rate of SA (r_SA_) is plotted against the dilution rate. The y-axis intersection on this graph gives the maintenance production rate, which was determined to be 0.3 g SA g^-1^ cells h^-1^. The maintenance production rate for the batch fermentation can be obtained by dividing the slope of the SA profile (Figure 3) by the DCW (once there is no further increase in the DCW). It is evident that the slope of the SA profile decreases towards the end of the fermentation. The maximum slope (and maximum maintenance production rate) was obtained right after growth termination, and was determined to be 0.6 g SA g^-1^ cells h^-1^. This is twice the rate of the chemostat estimate. The consequent decrease in SA production could not be attributed to the decrease in the DCW, implying that the r_SA_ decreases towards the end of the fermentation. Cell death might be a possible explanation for the observation.

## Conclusions

Tests on the modified *E. coli* KJ134 revealed that the flux distribution of the cell is not constant: higher SA fluxes were obtained under non-growth conditions, and SA yields in excess of 0.9 g g^-1^ are possible. Results from the continuous culture fermentations suggest that the optimal SA-producing pathway is absent during growth, with AcA production supplying the reduction requirements, similar to those of the wild SA producers. The intended metabolic pathway, where the TCA oxidative branch (or the glyoxylate shunt) supplies the reduction requirements, mainly functions under non-growth conditions, as observed from the batch fermentation.

Growth termination occurred close to a SA concentration of 18 g L^-1^ – the amount of AcA from the batch and chemostat fermentations was similar. This indicated that high titre (as well as high yield) production is only possible with non-growing cells. The maximum maintenance rate with SA production in the batch reactor was found to be twice that of the chemostat estimate.

## Methods

### Microorganism

The modified *E. coli* KJ134 strain was supplied by the Department of Microbiology and Cell Science of the University of Florida. The organism was genetically modified by the research group of Prof. L. Ingram [14]. Vials containing treated beads in a cryopreservative solution were used to store culture samples at -75°C [25]. All chemicals used in the fermentations were obtained from Merck KgaA (Darmstadt, Germany) unless otherwise specified. Temporary stock cultures were grown in Luria-Burtani broth. A defined medium named AM1, which was developed by Martinez et al. [26], was used in the fermentations and to grow the inoculum. The medium was supplemented with 50 g L^-1^ D-glucose, 100 mM KHCO_3_ and 100 mM MOPS before stock culture from the Luria-Burtani broth was added. It was then incubated at 37°C and 100 rpm over a period of 16–20 h in 30 mL sealed bottles containing 15 mL of the medium before the continuous and batch reactors were inoculated.

### Media composition

20 or 50 g L^-1^ glucose was added to the AM1 medium (excluding betaine hydrochloride (HCl)) for the continuous fermentations. Batch fermentations were carried out with initial glucose concentrations of 50 and 90 g L^-1^. Betaine HCl was used in the fermentation containing 90 g L^-1^ glucose, as recommended by Martinez et al. [26] for media containing glucose concentrations in excess of 50 g L^-1^. CO_2_ (g) (African Oxygen, Johannesburg, South Africa) was used as the inorganic carbon source.

### Continuous fermentations

The bioreactor setup employed an external recycle for agitation and is described in detail by van Heerden et al. [27]. Each reservoir was fitted with a 0.2 μm PTFE (polytetrafluoroethylene) membrane filter (Midisart 2000 filters from Sartorius, Göttingen, Germany). The working volume of the fermentations was 156 mL. The bioreactor consisted of an aluminium top and bottom section and a glass tube with a length of 115 mm and an inner diameter of 37.5 mm. The bottom section contained one entry for fermentation broth and also transferred heat from the hotplate to the broth. The top section contained a sealed aluminium sheath that acted as a thermowell for the thermocouple and two additional entry/exit points for the broth. The complete setup was autoclaved for 40 min at 121°C prior to starting the fermentation. The solution containing glucose and MgSO_4_ was autoclaved separately from the solution containing the rest of the salts to prevent precipitation and caramelisation of the sugar. The reactor was filled and operated at a temperature of 37°C and a pH of 7.00 ± 0.05 before it was seeded with approximately 8–10 mL of the inoculum. 10 M non-sterile potassium hydroxide (KOH) was used to control the pH. After startup the fermentor was operated at a dilution rate of 0.056 h^-1^ or lower. This was done to approach batch conditions to allow for initial accumulation of biomass. Continuous CO_2_ (g) flow into the recycle line of the reactor was controlled at 5–10 mL min^-1^ (approximately 0.05 vvm) with a Brooks thermal mass flow controller. Antifoam A (Sigma-Aldrich, St. Louis, USA) was added to the fermentation broth to control foam formation. Dilution rates between 0.021 and 0.187 h^-1^ were employed and run continuously in excess of five turnovers to approach steady state conditions. The stability and reproducibility of results were investigated by repeating dilution rates at different times in a fermentation and in fermentations started up separately.

### Batch fermentations

A 2 L Jupiter 2.0 fermentor (Solaris Biotechnology, Mantova, Italy) was used for the batch fermentations. A working volume of 1.5 L was used and operated at a temperature of 37°C and a pH of 7.00 ± 0.1. 10 M KOH was used for pH control. 50 mL of inoculum was injected into the reactor after the temperature and pH had stabilised. Antifoam A, diluted 1:9 with distilled water, was pumped into the fermentor as required. CO_2_ (g) was continuously sparged into the fermentor at approximately 0.3 L min^-1^ (approximately 0.2 vvm) before and during fermentations. Samples of 10–20 mL were extracted aseptically from the reactor.

### Analytical methods

Glucose and organic acid concentrations were determined by using high-performance liquid chromatography. An Agilent 1260 Infinity HPLC (Agilent Technologies, USA), equipped with an RI detector and a 300 × 7.8 mm Aminex HPX-87H ion-exchange column (Bio-Rad Laboratories, USA), was used for this purpose. The mobile phase (0.3 mL L^-1^ H_2_SO_4_) was fed at a flowrate of 0.6 mL min^-1^ with the column temperature at 60°C. The DCW was determined from 4.5–20 mL samples centrifuged at 750 g for 10 min. The cell pellets were washed twice with distilled water and dried at 90°C for at least 24 h.

## Abbreviations

AcA: Acetic acid; D: Dilution rate (h^-1^); DCW: Dry cell weight; FL: Formate lyase; Glu: Glucose; HCl: Hydrochloride; KOH: Potassium hydroxide; PDH: Pyruvate dehydrogenase; PEP: Phosphoenolpyruvate; PSA: Productivity (g L^-1^ h^-1^); rSA: Specific maintenance production rate of succinic acid (g g^-1^ h^-1^); SA: Succinic acid; TCA: Tricarboxylic acid; YSP: Succinic acid yield (g g^-1^); YSX: Cell yield (g g^-1^).

## Competing interest

The authors declare that they have no competing interests.

## Authors’ contributions

CDVH and WN contributed to the design of the experiments. CDVH executed the fermentations and other experimental work. WN did the interpretation, analysis and discussion of data. Both authors contributed towards writing the manuscript.

## References

[B1] Bomgardner MM Myriant to build succinic acid plant in Louisiana Chem Eng News 2011 89 7

[B2] Yuzbashev TV Yuzbasheva EY Laptev IA Sobolevskaya TI Vybornaya TV Larina AS Gvilava IT Antonova SV Sineoky SP Is it possible to produce succinic acid at a low pH? Bioeng Bugs 2011 2 115 119 10.4161/bbug.2.2.14433 21637000

[B3] Zeikus JG Jain MK Elankovan P Biotechnology of succinic acid production and markets for derived industrial products Appl Microbiol Biotechnol 1999 51 545 552 10.1007/s002530051431

[B4] Song H Lee SY Production of succinic acid by bacterial fermentation Enzyme Microb Technol 2006 39 352 361 10.1016/j.enzmictec.2005.11.043

[B5] McKinlay JB Vieille C Zeikus JG Prospects for a bio-based succinate industry Appl Microbiol Biotechnol 2007 76 727 740 10.1007/s00253-007-1057-y 17609945

[B6] Bechthold I Bretz K Kabasci S Kopitzky R Springer A Succinic acid: A new platform chemical for biobased polymers from renewable resources Chem Eng Technol 2008 31 647 654 10.1002/ceat.200800063

[B7] Cukalovic A Stevens CV Feasibility of production methods for succinic acid derivatives: a marriage of renewable resources and chemical technology Biofuels Bioprod Biorefin 2008 2 505 529 10.1002/bbb.105

[B8] Delhomme C Weuster-Botz D Kühn FE Succinic acid from renewable resources as a _C4_ building-block chemical—a review of the catalytic possibilities in aqueous media Green Chem 2009 11 13 26 10.1039/b810684c

[B9] Chimirri F Bosco F Ceccarelli R Venturello A Geobaldo F Succinic acid and its derivatives : fermentative production using sustainable industrial agro-food by-products and its applications in the food industry Ital J Food Sci 2010 22 119 125

[B10] Beauprez JJ De Mey M Soetaert WK Microbial succinic acid production: Natural versus metabolic engineered producers Process Biochem 2010 45 1103 1114 10.1016/j.procbio.2010.03.035

[B11] Cheng KK Wang GY Zeng J Zhang JA Improved succinate production by metabolic engineering BioMed Res Int 2013 1 12 10.1155/2013/538790 PMC3652112 23691505

[B12] Raab AM Lang C Oxidative versus reductive succinic acid production in the yeast *Saccharomyces cerevisiae* Bioeng Bugs 2011 2 120 123 10.4161/bbug.2.2.14549 21637001

[B13] Smidt M A sustainable supply of succinic acid Euro Biotech News 2011 10 C 11 12

[B14] Jantama K Zhang X Moore JC Shanmugam KT Svoronos SA Ingram LO Eliminating side products and increasing succinate yields in engineered strains of *Escherichia coli* C Biotechnol Bioeng 2008 101 881 893 10.1002/bit.22005 18781696

[B15] Hong SH Lee SY Importance of redox balance on the production of succinic acid by metabolically engineered *Escherichia coli* Appl Microbiol Biotechnol 2002 58 286 290 10.1007/s00253-001-0899-y 11935177

[B16] Vemuri GN Eiteman MA Altman E Succinate production in dual-phase *Escherichia coli* fermentations depends on the time of transition from aerobic to anaerobic conditions J Ind Microbiol Biotechnol 2002 28 325 332 10.1038/sj.jim.7000250 12032805

[B17] Isar J Agarwal L Saran S Saxena RK A statistical method for enhancing the production of succinic acid from *Escherichia coli* under anaerobic conditions Bioresource Technol 2006 97 1443 1448 10.1016/j.biortech.2005.07.014 16162408

[B18] Balzer GJ Thakker C Bennett GN San KY Metabolic engineering of *Escherichia coli* to minimize byproduct formate and improving succinate productivity through increasing NADH availability by heterologous expression of NAD(+)-dependent formate dehydrogenase Metab Eng 2013 20 1 8 23876411 10.1016/j.ymben.2013.07.005

[B19] Leib TM Pereira CJ Villadsen J Bioreactors: a chemical engineering perspective Chem Eng Sci 2001 56 5485 5497 10.1016/S0009-2509(01)00173-7

[B20] Lübbert A Jørgensen SB Bioreactor performance: a more scientific approach for practice J Biotechnol 2001 85 187 212 10.1016/S0168-1656(00)00366-7 11165363

[B21] Villadsen J Innovative technology to meet the demands of the white biotechnology revolution of chemical production Chem Eng Sci 2007 62 6957 6968 10.1016/j.ces.2007.08.017

[B22] Shuler M Kargi F Bioprocess Engineering. Second Edi 2002 New Jersey: Prentice Hall

[B23] Villadsen J Nielsen J Lidén G Principles BE Bioreaction Engineering Principles. Third Edit 2011 New York: Springer

[B24] Jantama K Haupt MJ Svoronos SA Zhang X Moore JC Shanmugam KT Ingram LO Combining metabolic engineering and metabolic evolution to develop nonrecombinant strains of *Escherichia coli* C that produce succinate and malate Biotechnol Bioeng 2008 99 1140 1153 10.1002/bit.21694 17972330

[B25] Pro-Lab Diagnostics Microbank™ 2011 Neston, UK: Worldwide Performance Portfolio 1 78

[B26] Martinez A Grabar TB Shanmugam KT Yomano LP York SW Ingram LO Low salt medium for lactate and ethanol production by recombinant *Escherichia coli* B Biotechnol Lett 2007 29 397 404 10.1007/s10529-006-9252-y 17160622

[B27] Van Heerden CD Nicol W Continuous succinic acid fermentation by *Actinobacillus succinogenes* Biochem Eng J 2013 73 5 11

